# See–saw relationship of the Holocene East Asian–Australian summer monsoon

**DOI:** 10.1038/ncomms12929

**Published:** 2016-09-26

**Authors:** Deniz Eroglu, Fiona H. McRobie, Ibrahim Ozken, Thomas Stemler, Karl-Heinz Wyrwoll, Sebastian F. M. Breitenbach, Norbert Marwan, Jürgen Kurths

**Affiliations:** 1 Potsdam Institute for Climate Impact Research (PIK), 14473 Potsdam, Germany; 2 Department of Physics, Humboldt University, 12489 Berlin, Germany; 3 School of Earth and Environment, The University of Western Australia, Crawley, Western Australia 6009, Australia; 4 Department of Physics, Ege University, 35100 Izmir, Turkey; 5 School of Mathematics and Statistics, The University of Western Australia, Crawley, Western Australia 6009, Australia; 6 Sediment- and Isotope Geology, Institute for Geology, Mineralogy & Geophysics, Ruhr-Universität Bochum, Universitätsstr. 150, 44801 Bochum, Germany; 7 Institute for Complex Systems and Mathematical Biology, University of Aberdeen, Aberdeen AB24 3UE, UK

## Abstract

The East Asian–Indonesian–Australian summer monsoon (EAIASM) links the Earth's hemispheres and provides a heat source that drives global circulation. At seasonal and inter-seasonal timescales, the summer monsoon of one hemisphere is linked via outflows from the winter monsoon of the opposing hemisphere. Long-term phase relationships between the East Asian summer monsoon (EASM) and the Indonesian–Australian summer monsoon (IASM) are poorly understood, raising questions of long-term adjustments to future greenhouse-triggered climate change and whether these changes could ‘lock in' possible IASM and EASM phase relationships in a region dependent on monsoonal rainfall. Here we show that a newly developed nonlinear time series analysis technique allows confident identification of strong versus weak monsoon phases at millennial to sub-centennial timescales. We find a see–saw relationship over the last 9,000 years—with strong and weak monsoons opposingly phased and triggered by solar variations. Our results provide insights into centennial- to millennial-scale relationships within the wider EAIASM regime.

## Abstract (web summary)

Irregular sampling of palaeoproxies can result in the misinterpretation of environmental records. Here, the authors propose a new time series analysis method for irregularly sampled data, and reveal a see-saw relationship between the East Asian and Indonesian–Australian summer monsoons during the Holocene.

## Graphical abstract

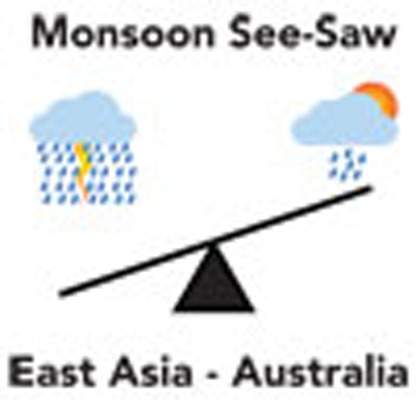

High-resolution speleothem proxy records from cave KNI-51 (15.30° S, 128.61° E) in northwestern Australia and Dongge Cave (DA) (25.28° N, 108.08° E) from southern China (Fig. 1) provide an outline of the summer monsoon states of the last 9,000 years^1^. Details of the U/Th chronology and stable isotope records are given by Denniston *et al.*^2^ and Wang *et al.*^3^, respectively. Both caves are well placed to capture the summer monsoon regimes located at the end points of the EAIASM system (Fig. 1 and Supplementary Fig. 2 as well as Supplementary Discussion). Stalagmite *δ*^18^O time series have prominently been used to identify and study past changes in summer monsoon strength^4^. The *δ*^18^O signal recorded in Asian stalagmites depends on multiple factors, including moisture source composition and distance, Rayleigh fractionation during moisture transport, and amount of precipitation. These factors, and thus stalagmite *δ*^18^O, are all directly related to summer monsoon strength^2^,^5^,^6^,^7^,^8^,^9^. A more distal moisture source lengthens the transport pathway to the study site, and Rayleigh distillation during rainout, which in turn leads to more negative *δ*^18^O in monsoonal rainfall and associated infiltrating and drip water, ultimately resulting in more negative stalagmite *δ*^18^O. Thus, speleothem *δ*^18^O is a complex integral of multiple factors, not exclusively reflecting local rainfall amount, but instead providing a valid proxy for monsoon strength in a more general sense^4^,^10^. In some instances a pronounced amount effect is observed. For example, in the IASM realm, rainfall *δ*^18^O is mainly linked to rainfall amount, as a comparison of rainfall amount and *δ*^18^O at the Global Network of Isotopes in Precipitation (GNIP) station at Darwin (∼400 km SW of KNI-51) demonstrates (*R*^2^=0.8; *P*<0.001)^2^. Positive *δ*^18^O excursions in stalagmites coincident with the timing of graffiti on cave walls telling of massive droughts, exemplify the sensitivity of *δ*^18^O to drought in the EASM region^7^. A negative example was found in NE India, where the amount effect is clearly absent^5^, but speleothem *δ*^18^O still records changes in Indian summer monsoon strength linked to ENSO^11^. Thus, we emphasize again that the *δ*^18^O variability acts as a proxy for monsoon strength and not rainfall amount alone. Moreover, advanced nonlinear time series analysis methods can be used to analyse the dynamical imprint of the monsoon activity in the *δ*^18^O record and by analysing the time series it is possible to go beyond an interpretation of just the values of *δ*^18^O.

The records of DA and KNI-51 are irregularly sampled, that is, the time between two consecutive measurements is not constant and may vary largely along the length of the record. Most time series analysis methods, however, require regular sampling. Traditionally, some form of interpolation is used to deal with these irregularities, but this introduces additional information into the time series with much higher uncertainty than the real observations^12^. To avoid corrupting the quality of the proxy records, a newly developed method can be used (see refs 13, 14) that is based on techniques used for the neurological data^15^. This Transformation Cost Time Series (TACTS) method produces a detrended and regularly sampled time series, that can be further analysed with standard time series analysis methods to identify regime changes.

Here we show that the TACS method is well suited to analyse the records of DA and KNI-51 and can detect statistical significant dynamical details of the monsoon dynamics by distinguishing phases of strong/weak monsoon on centennial time scale. This allows us to substantiate and improve previous more qualitative interpretations of the DA and KNI-51 records^2^,^3^,^16^. Overall, the phase relationship between major regime shifts in the two records is anticorrelated (see Figs 2 and 3). Phases of strong (weak) monsoon activity in the northern hemisphere (DA proxy) coincide with phases of weak (strong) monsoon activity in the southern hemisphere (KNI-51 proxy). Solar activity provides a likely driver of this see–saw dynamics and our analysis confirms previous conclusions that solar activity can impact on the overall monsoon dynamics by shifting the position of the the Intertropical Convergence Zone (ITCZ)^3^.

## Results

### Transitions of the monsoon activity

**Figure 2:**
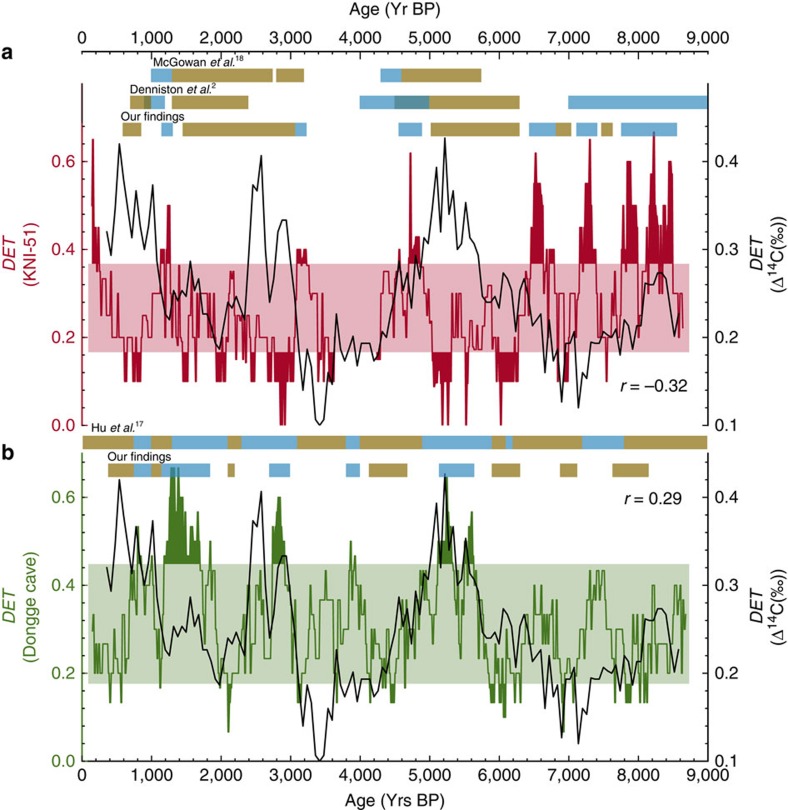
Determinism of the KNI-51 Cave and Dongge Cave records with comparison to previous studies. (**a**) (red) KNI-51 cave and (**b**) (green) Dongge Cave (DA). The determinism is calculated from the corresponding transformation costs time series and statistical significance is indicated by the two horizontal bands (see Methods section for details). High (low) determinism values correspond to wet (dry) monsoon regimes. The coloured bands (blue indicating wet regimes; brown, dry) provide a comparison of our findings with those of previous, qualitative studies. In the text we provide a detailed discussion of previously unidentified or incorrectly identified wet and dry regimes uncovered by our method. (black) Determinism of the solar activity proxy Δ^14^C time series. Cross-correlation between the determinism of the solar activity proxy Δ^14^C time series and KNI-51 time series is *r*=−0.32, and DA time series is *r*=0.29 (see Methods section for details).

Analysis of the DA and KNI-51 records reveals alternating periods of statistically significant centennial to millennial-scale strong/weak monsoon states (Fig. 2). Strong/weak states are defined as exceeding the confidence bands. Prolonged strong/weak states are identified, and the comparison given by the coloured bands in Fig. 2 highlights that our quantitative technique is able to reveal new details of the monsoon dynamics.

The strong/weak regimes identified improve upon previous, qualitative interpretations of the proxy records^2^,^3^,^16^. Here we provide a detailed discussion of where our method supports, corrects and improves earlier studies. We particularly focus on regimes which are newly identified or previously incorrectly interpreted.

Major strong(weak) phases, defined in kiloyears Before Present (ka BP), occur in the northwest Australian summer monsoon domain between 8.5–6.4 ka BP (6.3–5.0 ka BP), 5.0–4.0 ka BP, possibly extending to 3.0 ka BP, (3.0–1.4 ka BP), 1.3–0.9 ka BP, with a transition at 0.9 ka BP to the present regime.

Embedded within these time intervals are additional events of centennial to sub-centennial duration. Unfortunately, the details of the Holocene summer monsoon of northwestern Australia are largely unknown, precluding any comparison of stratigraphic records. Nevertheless, a recent pollen-sediment record from Black Springs (northwestern Kimberley)^17^ shows some correspondence to our phase record, but the pollen record is poorly resolved, supported by only four radiocarbon dates. Our analyses offers improved time resolution and greater details of the inherent variability within major monsoon phases.

### Cross-hemispheric see–saw dynamics

The Dongge Cave record has been discussed by Wang *et al.*^3^, further developed by Hu *et al.*^16^ and more recently by Zhao *et al.*^18^. Wang *et al.*^3^ recognised eight weak monsoon events lasting 100 to 500 years: at 0.5 ka BP, 1.6 ka BP, 2.7 ka BP, 4.4 ka BP, 5.5 ka BP, 6.3 ka BP, 7.2 ka BP and 8.3 ka BP. While adding some details, the Hu *et al.*^16^ reconstructions essentially concur with those of Wang *et al.*^3^. Our results indicate strong/weak regime intervals between (8.2–7.6 ka BP), 7.6–7.2 ka BP, (7.1–6.9 ka BP), (6.4–5.8 ka BP), 5.8–5.0 ka BP, (5.0–4.0 ka BP), 3.0–2.7 ka BP, (2.2–2.0 ka BP), 1.9–0.8 ka BP and (0.7–0.4 ka BP). A comparative study applying our method on the palaeo Summer Monsoon Index (SMI) derived from sediments of the Qinghai Lake^19^ corroborates our findings (see Supplementary Discussion and Supplementary Fig. 4 for details).

Our analysis has revealed details for KNI-51 and DA not previously recognized (Fig. 2). In the KNI-51 record two events, absent from Denniston *et al.*^2^, occur at 6.6–6.4 ka BP (weak monsoon/wet) and 7.0–6.8 ka BP (strong monsoon/dry). Furthermore, our results improve upon the findings of Denniston *et al.*^2^ and McGowan *et al.*^17^ by reclassifying previously misinterpreted regimes. We identify a strong (wet) monsoon regime at 3.2–3.1 ka BP previously interpreted as dry^17^ and similarly a weak (dry) regime at 7.6–7.5 ka BP incorrectly claimed to be wet by Denniston *et al.*^2^.

Similarly, the results of our DA analysis contradict the conclusions of Hu *et al.*^16^ for the time periods 6.2–6.1 ka BP (weak) and 7.8–7.6 ka BP (weak). In addition, there are three events identified by Hu *et al.*^16^ that are not statistically significant in our analysis (3.4–3.2 ka BP, 6.9–6.3 ka BP and 8.8–8.2 ka BP). We assert confidence in these revisions, as they are based on a rigorous, quantitative analysis, rather than rudimentary visual comparison of data sets. The detailed comparison of our findings and the literature summary is given in Supplementary Tables 1 and 2.

**Figure 3:**
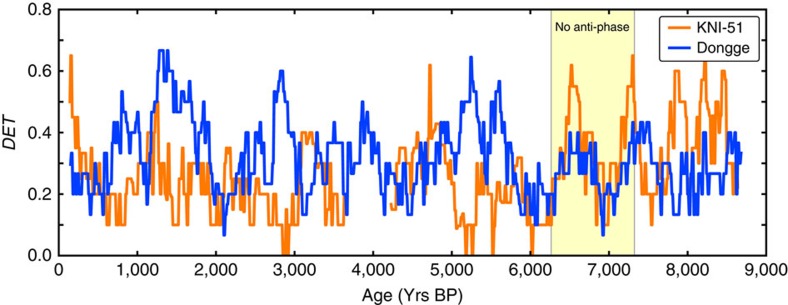
Determinism of KNI-51 and Dongge Cave highlighting the antiphase relationship. There is a gap in the data of KNI-51 around ∼4,000 yr BP. Contrary to the general antiphase relation of the two determinism time series of KNI-51 and Dongge Cave proxies, the region around ∼7,000 yr BP shows a in—phased relationship, highlighted with yellow.

Moreover, our results reveal a striking strong/weak, opposing relationship between the IASM^2^ and EASM^3^ (Fig. 2). The only time when this see–saw relationship is not observed is during 7.6–7.2 ka BP, when both monsoon records show a ‘weak state'. Over the entire time scale, the cross-correlation of the DET time series is −0.27, and while this affirms an antiphased relationship, it does not capture the strong correspondence between the statistically significant strong/weak monsoon states. In fact the antiphased relationship is much stronger, if only the statistical significant parts of the time series are used and the internal variability on sub-centennial to decadal time scales is ignored. This may be calculated using a step function filter, yielding a cross-correlation of −0.33. This can be perceived by simultaneous plot of DET values for KNI-51 and DA in one figure (Fig. 3). Comparable results are found in the Qinghai Lake data (SMI) with a cross-correlation of −0.28 (Supplementary Fig. 4). Therefore, the variability at sub-centennial to decadal time scales in both the DA and KNI-51 records is emphasized; such short-term variability is evident in present-day monsoon records from both regions^20^.

### Impact of solar activity on monsoonal see–saw pattern

While the details of the controls and processes determining the function and latitudinal extent of the respective summer monsoons are more complex^21^,^22^ than simply relating them to the position of the ITCZ, still the ITCZ provides a convenient metric of monsoon extent^21^,^23^,^24^. For the broader EAIASM history, the displacement of the ITCZ is a driver that has been advocated in a range of Quaternary paleoclimate studies^25^,^26^,^27^,^28^,^29^. The argument recognises that the ITCZ is displaced towards the warmer hemisphere in response to differential cooling^30^,^31^,^32^. This is an attractive and apparently straightforward explanation, with a caveat that the ITCZ over the region of the West Pacific Warm Pool (that is, the Maritime Continent) is much less well defined than over the wider Pacific and Indian Oceans, with a more complex south-north (north–south) seasonal migration pattern^23^,^33^,^34^.

In explaining the DA *δ*^18^O record, Wang *et al.*^3^ appeal to a likely displacement of the ITCZ driven by solar variability. They use the atmospheric Δ^14^C record^35^ as a proxy for solar activity with which they obtain a correlation of 0.3 with their speleothem *δ*^18^O record. The inference is followed by Zhao *et al.*^18^ who support the claim of a concordance of the DA *δ*^18^O record with solar variability. We extend this claim further and ask whether the Holocene antiphase relationship that we have uncovered in the summer monsoons of the overall EAIASM is driven by solar variability.

To establish this, we compare the determinism-measure of solar activity with that derived from the EASM and IASM proxy records. The analysis identifies a statistically significant correlation between solar activity and both records from DA with correlation of 0.29 and KNI-51 with correlation of −0.32 (SMI: 0.35; see Supplementary Fig. 4). Thus, when predictability of solar activity is high (low), the Dongge Cave record indicates a strong (weak) summer monsoon, while northern Australia experiences a weak (strong) summer monsoon. Increased predictability of solar activity corresponds to periods of a consistently high number of solar ‘events', increasing the solar irradiance received by the Earth. Positive correlation with the Dongge Cave record therefore indicates a direct control, whereby periods of increased solar activity enhance the summer monsoon over East Asia. The asymmetric response in the Australian monsoon record suggests that periods of increased solar irradiance actually decrease monsoon strength. To explain this, we consider orbital-scale positioning of the ITCZ. Preferential heating of the Northern Hemisphere during periods of high tilt and Northern Hemisphere perihelion, as observed from 9 to 3 ka, provides a background driver for increased EASM strengthening. At a global scale, there is a northward shift in the ITCZ, weakening monsoon activity over north west Australia. Coupling this shift with solar activity, brief periods of increased irradiance would act to shift the ITCZ further north, and we would therefore expect a stronger EASM and corresponding weak IASM. This mechanism is supported by our analysis, and compounded by the observation that from c.2.5 ka onwards, as orbital controls begin to favour the Southern Hemisphere, correspondence between the determinism-measure of solar activity and EASM and IASM records diminishes. These findings lead us to conclude that solar activity provides a driver in the see–saw relationship observed between the EASM and IASM over the past 9,000 years, modulated by orbital-scale ITCZ positioning.

## Discussion

We note that in our interpretation we cannot rule out the likelihood of ENSO events playing a role. Mann *et al.*^36^ using the Zebiak–Cane model of the tropical Pacific ocean-atmosphere system demonstrated that changes in solar radiative forcing provokes an El Niño response. However, the impact of ENSO events on both monsoon regimes is complex and difficult to disentangle. Summer rainfall records from the NW Australian monsoon region lack a significant ENSO signature (http://bom.gov.au/climate/enso/ninocomp.shtml). On the other hand, the Southern Oscillation Index, SOI, has been shown to influence this region^37^, where a likely impact can be claimed only for very strong negative/positive SOI values. In contrast, the EASM is clearly influenced by ENSO^11^,^38^,^39^,^40^,^41^, but with regional (north–south) differences^40^, complex phase-modulation relationships^38^,^42^ and with specific ENSO events having quite a different regional rainfall expression—for example, the 1997/1998 and 1982/1983 events. The role of ENSO is an open question and the lack of well-expressed significant variation of the ENSO during the last 7,000 years makes it difficult to answer it (ref. 43). These facts, finally, do not allow to infer a clear ENSO driving of the antiphase relationship between the IASM and the EASM at the Holocene time scale.

A significant body of work is now available that highlights the impact of solar variability on the tropical atmosphere^44^,^45^,^46^,^47^. This work demonstrates that the Hadley and Walker circulation are affected by solar variability, and through this, trigger an increase in tropical precipitation during periods of high solar activity and an associated change in the position of the ITCZ. Thus, solar variability can force the north–south expansion and contraction of the ITCZ over the region of the East-Asian–Indonesian–Australian–Monsoon region^48^. We demonstrate that solar variability can impact summer monsoon strength, and more importantly provides the control of the antiphase relationship between the EASM and IASM over the last 9,000 years. Our results reveal a strong coupling between the monsoons of the two hemispheres, expressed as a seesaw relationship, and driven by decadal to centennialscale variations in solar activity. A full understanding of how solar variability can drive the monsoon response requires focused model studies. From these will emerge the likelihood of disentangling the overall functioning of the EAIASM regime, forming a further step in understanding how this regime will respond to present-day Greenhouse forcing, which may help to secure the future of people living in the region.

## Methods

### TACTS method

In essence, the TACTS method determines the ‘cost' of transforming one segment of a record into the following segment. For this transformation we allow three possible modifications: first changing the amplitude of a data point, second shifting a data point in time, and third creating or deleting a data point. The ‘cost' for changing the amplitude and shifting a data point is linearly dependent on the size of the modification. However, creating and deleting data points should be ‘expensive' enough to not favour this modification over the other two points.

Many time series, for example, palaeoclimate proxy records, show cumulative trends, which usually need to be removed in a preprocessing step before time series analysis. A common procedure for regularly sampled time series is to apply a difference detrending filter, Δ*x*=*x*(*t*)−*x*(*t*−1), simply taking the difference between consecutive points. The TACTS method is a similar approach for detrending but for irregularly sampled time series. Here the difference between subsequent sequences is expressed by an associated transformation cost as explained below (see Supplementary Discussion for details).

To calculate the transformation cost time series, we determine the cost for transformation of one segment into another for two successive segments of a time series. Treating each observation as an ‘event', we seek to transform the events in the first segment into those of the second. For a single transformation, this cost is a generalized distance between these two segments. Therefore, as a distance, the cost must be a positive number, symmetrical (that is, transforming the first into the second is the same as transforming the second into the first), and must satisfy the triangle inequality.

The cost associated with each transformation is given by:


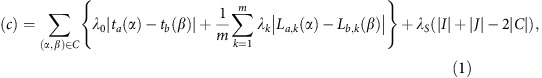


where *I* and *J* are a set of indices of the events in starting set *S*_*a*_ and the final set *S*_*b*_, respectively. These sets—*S*_*a*_ and *S*_*b*_—correspond to the events in the two time series segments. The first summation quantifies the cost associated with shifting events in time. We sum over the pairs (*α*, *β*)∈*C*, where the set *C* comprises the points that need to be shifted in time. *α* and *β* denote the *α*th event in *S*_*a*_ and *β*th event in *S*_*b*_. The coefficient *λ*_0_ is the cost factor for time shifts. The second summation calculates the cost due to changing the amplitude of events. This involves the difference |*L*_*a*,*k*_(*α*)−*L*_*b*,*k*_(*β*)|, where *L*_*a*,*k*_(*α*) is the amplitude of the *α*th event in *S*_*a*_. The parameter *λ*_*k*_ has the unit of per amplitude and the sum is over the different components of the amplitude. That is, if we are dealing with one dimensional data *m*=1, while for a three dimensional phase space *m* would be three. The last terms in the cost function deal with the events not in *C* which have to be added or deleted. Note that |·| denotes the size of the set and *λ*_*S*_ is the cost parameter for this operation. Suzuki *et al.* omitted this parameter, since they chose a cost of one for such an operation^14^.

We determine the cost factors *λ*_0_, *λ*_*k*_ based on the time series at hand:


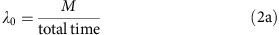



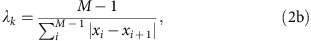


where *x*_*i*_ is the amplitude of *i*th element and *M* is the total number of events in the time series. Note that *λ*_0_ is the mean event frequency and *λ*_*k*_ is the inverse of the average amplitude difference.

The cost factor *λ*_*S*_ is an optimization parameter. We constrain *λ*_*S*_∈[0, 4] and explore the costs of deleting or adding an event to our time series. If our time series consists of *n*+1 segments of equal length, we can calculate *n* costs for each individual transformation of the segments. Assuming that the costs are linearly independent, the central limit theorem indicates that the distribution of the costs should be a normal distribution. In particular, when dealing with non-stationary data we find that changing *λ*_*S*_ such that the distribution becomes normal greatly improves the skill of our time series analysis method.

**Figure 4:**
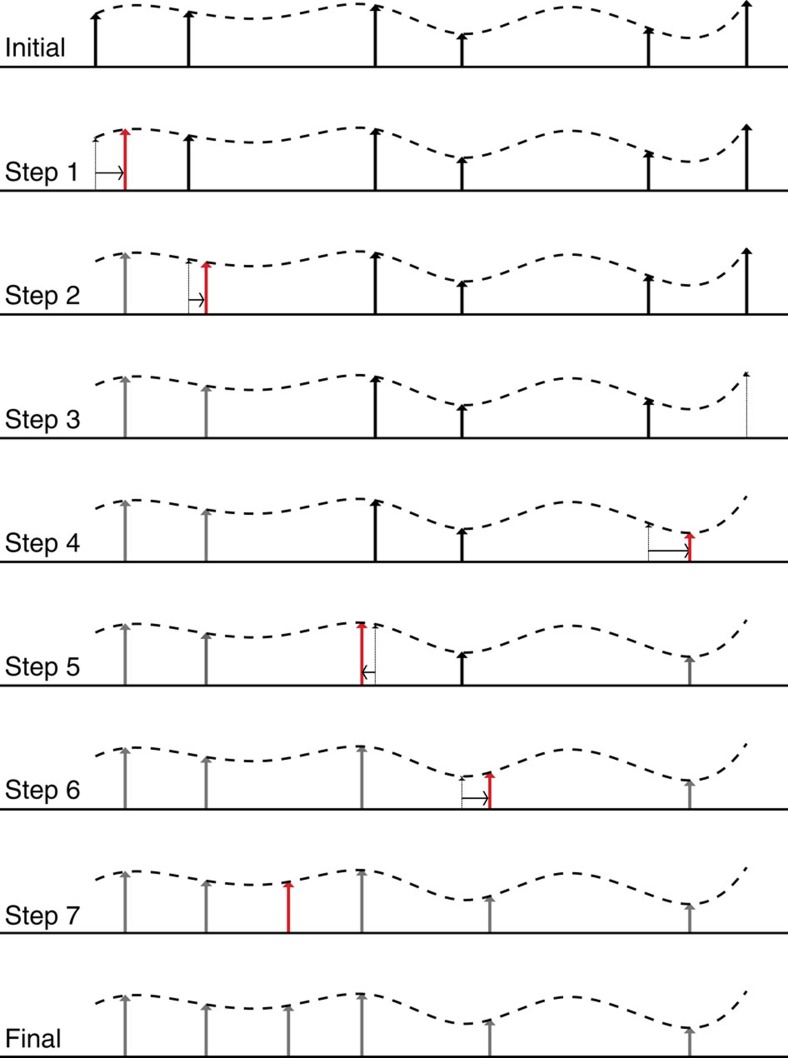
Illustration of the transformation cost time series method. The true time series from which the two time series are sampled is indicated by the dashed line. The initial time series segment (top) is transformed into the final time series segment (bottom) in seven steps. Note that after seven steps the segment is identical to the final target time series. The steps 1, 2, 4, 5, 6 are combinations of the elementary operations (i) time shift and (ii) adjusting the amplitude (first two terms of equation (3)), while in step 3 one event is deleted and therefore the (iii) elementary operation was applied (last term of equation (3)).

In Fig. 4, we give an illustration of how to perform this transformation. Recall that the transformation is done by three elementary steps: (i) shifting an event in time; (ii) changing the amplitude of the event; and (iii) creating or deleting an event. The figure outlines the steps required to transform the top time series segment into the bottom one. This transformation consists of seven elemental steps. Moves 1 and 2 move the first and second event to the right and, in addition, adjust their magnitude, that is, a combination of the two elementary steps (i) and (ii). In move three the last event is deleted (that is, elementary step (iii)). As we can see it takes four additional elementary steps (combinations of (i) and (ii)) to transform the starting time series into the target time series.

### Recurrence plot analysis

The resulting regularly sampled cost time series is analysed using recurrence plot analysis to derive the recurrence quantification measure determinism (DET)^49^. DET is a measure of predictability well suited to detect regime changes in time series. DET characterizes a specific, recurrence-based dynamical property, independent of the state of the system (that is, the amplitude of the *δ*^18^O at a given time). Therefore, DET values are not directly related to a specific state value such as strong or weak monsoon regime). Nevertheless, it is possible that certain regime states (for example, a strong monsoon regimes) are linked to a characteristic recurrence pattern, for example, a more regular and periodic dynamics (enhanced monsoon regimes) or less periodic and less predictable dynamics (weak monsoon or monsoon failure). Such relationships between states and recurrence properties seem typical in the climate system, where, for example, cooling events have been linked to less predictable (less regular) climate dynamics^13^,^50^. Depending on geographic location and regional climate mechanisms, such relationships can differ significantly and can even be opposite. On the basis of information from literature, we are able to link the characteristic dynamical property of determinism to a certain climate regime, such as dry or wet, for the considered proxy records.

For each proxy record, the transformation cost time series is divided into segment sizes of 20 years containing, on average, 4 to 5 points. The final results shown in Fig. 2 are relatively insensitive to the choice of segment size. The proportionality parameters for modifications (i) and (ii) are determined from the proxy records and are related to the average amplitude and sampling time. The creation and deletion cost factor *λ* is our optimisation parameter, chosen relative to the other parameters. Determining the costs of transformation provides a measure of how close one segment is to the following one and produces a regularly sampled transformation cost time series with a temporal resolution of 20 years. Using recurrence plot analysis, as described below, we are able to quantify the predictability of each segment by deriving the determinism^49^. Abrupt transitions into or out of a ‘wet' or ‘dry' state are hard to predict, while behaviour within a regime follows a somewhat similar pattern throughout. As a result, determinism is particularly effective at identifying regime changes.

Recurrence plots visualise a fundamental property of dynamical systems—namely, when a the system ‘repeats' itself, returning to a previous state. Formally, for a set of observations 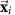 for *i*=1, …, *N* this is defined as


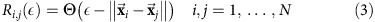


where 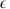 is some threshold distance, ||·|| is some distance measure, and Θ(*y*)=1 if *y*≥0 and 0 otherwise^49^. This method is well suited to capture regime changes, as such an extreme event would result in a sudden reduction in the number of recurring events. Plotting this matrix allows visual analysis of the system, and from this quantitative measures can be derived. Diagonal structures within the plot, running parallel to the main diagonal (bottom left to top right), indicate sections of the trajectory with locally similar paths. Calculating the fraction of points in the recurrence plot that form diagonal lines with respect to all points gives us the measure determinism. This is a measure of the amount of predictability within the system, as stochastic or chaotic systems result in none or only short diagonals. For the selected threshold distance 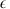, a histogram of diagonal lengths, *P*(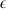, *l*), is derived and a minimal diagonal length, *l*_min_, is set. Determinism is then given by


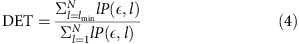


In this analysis, the recurrence plot is derived using the Euclidean distance norm and 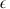-threshold distance is chosen adaptively to ensure a sensible density of ‘ones' in the RP, fixed at 10%. In the determinism calculation, we take *l*_min_ to be 2. These parameters were selected to ensure a balance between stability and, particularly in the case of the threshold distance, the inclusion of enough data points for the recurrence structure of the underlying system to be captured. For details on the embedding required to transform time series data into a trajectory in phase space see ref. 51.

The variation of a quantitative recurrence measure, such as DET, has to be tested whether its change is significant or not. We follow the approach by Marwan *et al.* and apply a bootstrapping technique^52^. The basic idea is that the dynamics of the system does not change over time. Such a change is usually measured by a sliding window approach, where DET_*i*_ means the recurrence measure calculated in the *i*th window. Within these windows *i*, we also have the histograms of diagonal lengths, *P*_*i*_(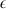, *l*). We now bootstrap the lengths *l* from the histograms of all windows and use these lengths to calculate DET of this bootstrapped histogram, allowing an average picture of the DET measure for the whole time. Repeating this procedure *N* times, we get an empirical test distribution for DET. The 5 and 95% quantiles are used to infer confidence (90%) about the variation of DET and allows us to judge whether the found variability of the measure DET is significantly different from an unchanged dynamics (that is, whether a regime transition occurs)^52^.

### Cross-correlation of two irregularly sampled time series

To compare the KNI-51 and Dongge Cave records with solar variability (see Supplementary Fig. 1a,b), we correlate these records with the atmospheric Δ^14^*C* record compiled by Stuiver *et al.*^53^ (see Supplementary Fig. 1c). This record, spanning 9,700 years, was compiled from radiocarbon tree ring ages and is a widely used proxy for solar irradiance with lower Δ^14^*C* values inferring increased solar irradiance^3^. This record is already sampled at regular time intervals so we do not need to apply the transformation cost function. However, the time steps of this data set do not align with the determinism time series generated from the speleothem records. We cannot, therefore, calculate cross-correlation without transforming the data sets again.

Interpolation is commonly used in such a scenario, but this creates artificial, and necessarily uncertain, data points in the time series. A Gaussian kernel-based cross-correlation (gXCF) has been demonstrated to reduce such biases relative to linear interpolation, as well as Lomb-Scargle, rectangular and quasi-sinusoidal kernel-based cross-correlation estimators^12^. We therefore use gXCF as our estimator of the similarity between the speleothem and solar activity data sets.

The benefit of kernel based techniques is that, rather than introducing new data to the time series, the two data sets are ‘matched' using a weighting function. Pearson cross-correlation takes the sum of the product of paired data points in two time series *X* and *Y*. However, using the kernel, each data point in time series *X* is multiplied by every data point in *Y*, but with a weighting function dependent on the distance between the time that these observations occurred. Kernel-based cross-correlation is therefore given by


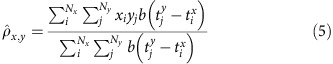


where 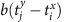 is the kernel, determining how much weight to give to the product of two observations *x*_*i*_ and *y*_*j*_, based on the time gap between them.

In the case of gXCF, the kernel is


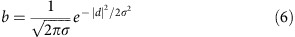


where *d* is the distance between the observation times 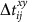 and *σ* is the s.d. of the kernel distribution, which scales the kernel. As there is no theory detailing the best choice of scaling parameter *σ*, we use *σ*=Δ*t*^*xy*^/4 as per Rehfeld *et al.*^12^.

### Data availability

The proxies from Dongge cave (*δ*^18^O) and solar activity (*δ*^14^C) are published/available data sets. *δ*^18^O proxy from KNI-51 cave, Kimberley is available from the authors. Requests for the TACTS of proxies can be sent to D.E. (eroglu@pik-potsdam.de).

## Additional information

**How to cite this article:** Eroglu, D. *et al.* See–saw relationship of the Holocene East Asian–Australian summer monsoon. *Nat. Commun.* **7,** 12929 doi: 10.1038/ncomms12929 (2016).

## Supplementary Material

Supplementary Information Supplementary Figures 1-4, Supplementary Tables 1-2, Supplementary Discussion and Supplementary References

## Figures and Tables

**Figure 1:**
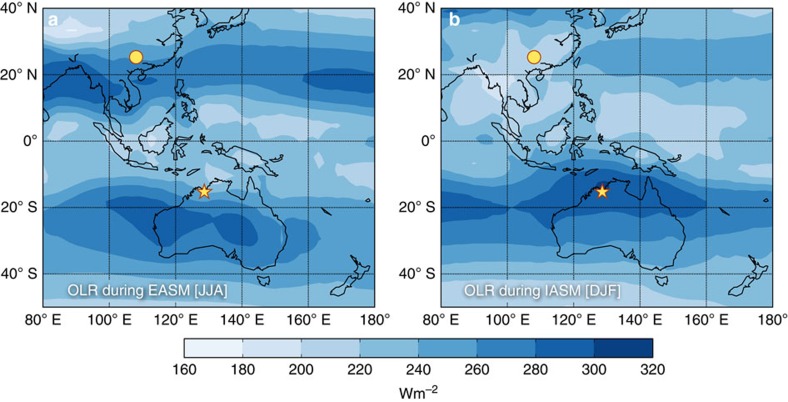
Top of atmosphere outgoing long wave radiation during the monsoon months delimiting its extent. (**a**) East Asian summer monsoon (EASM) during June, July and August (JJA); and (**b**) Indonesian–Australian summer monsoon (IASM) during December, January and February (DJF)^54^; Dongge Cave (dot) and KNI-51 cave (star).
