# Supplementary material for: See–saw relationship of the Holocene East Asian–Australian summer monsoon
Source: Nat Commun. 2016 Sep 26;7:12929. doi: 10.1038/ncomms12929 (PMC5052686; doi:10.1038/ncomms12929)
Supplement: Supplementary Information — Supplementary Figures 1-4, Supplementary Tables 1-2, Supplementary Discussion and Supplementary References [file ncomms12929-s1.pdf]

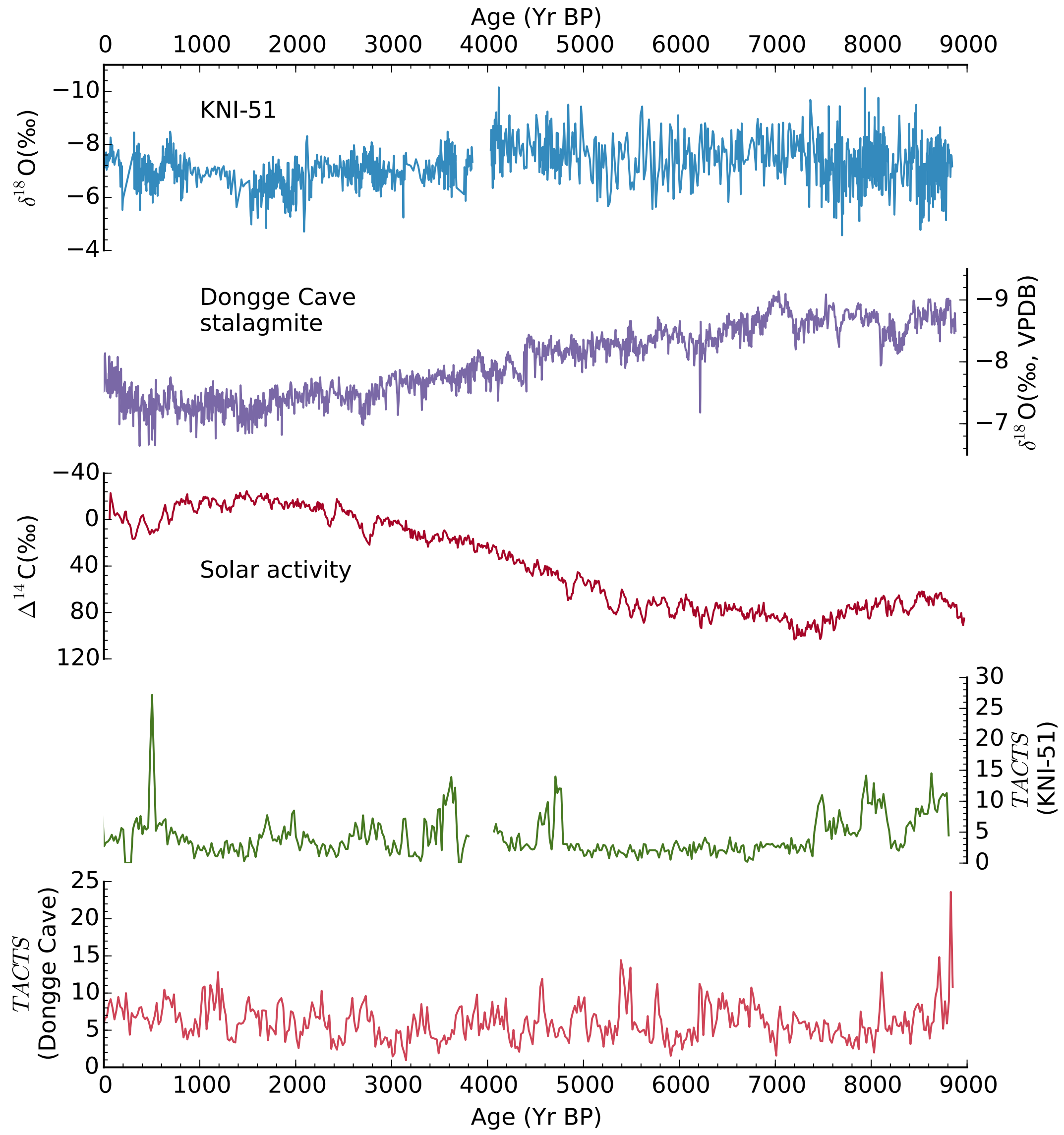

Supplementary Figure 1: **The proxies in the manuscript and their transformation cost time series.** δ^{18} O records of (a) KNI-51 and (b) Dongge Cave. (c) atmospheric $\Delta^{14}C$ record compiled by Stuiver et al. [1] and the transformation cost time series of (d) KNI-51 and (e) Dongge Cave determined by application of eq. (1) and (2) (equations in the main text).

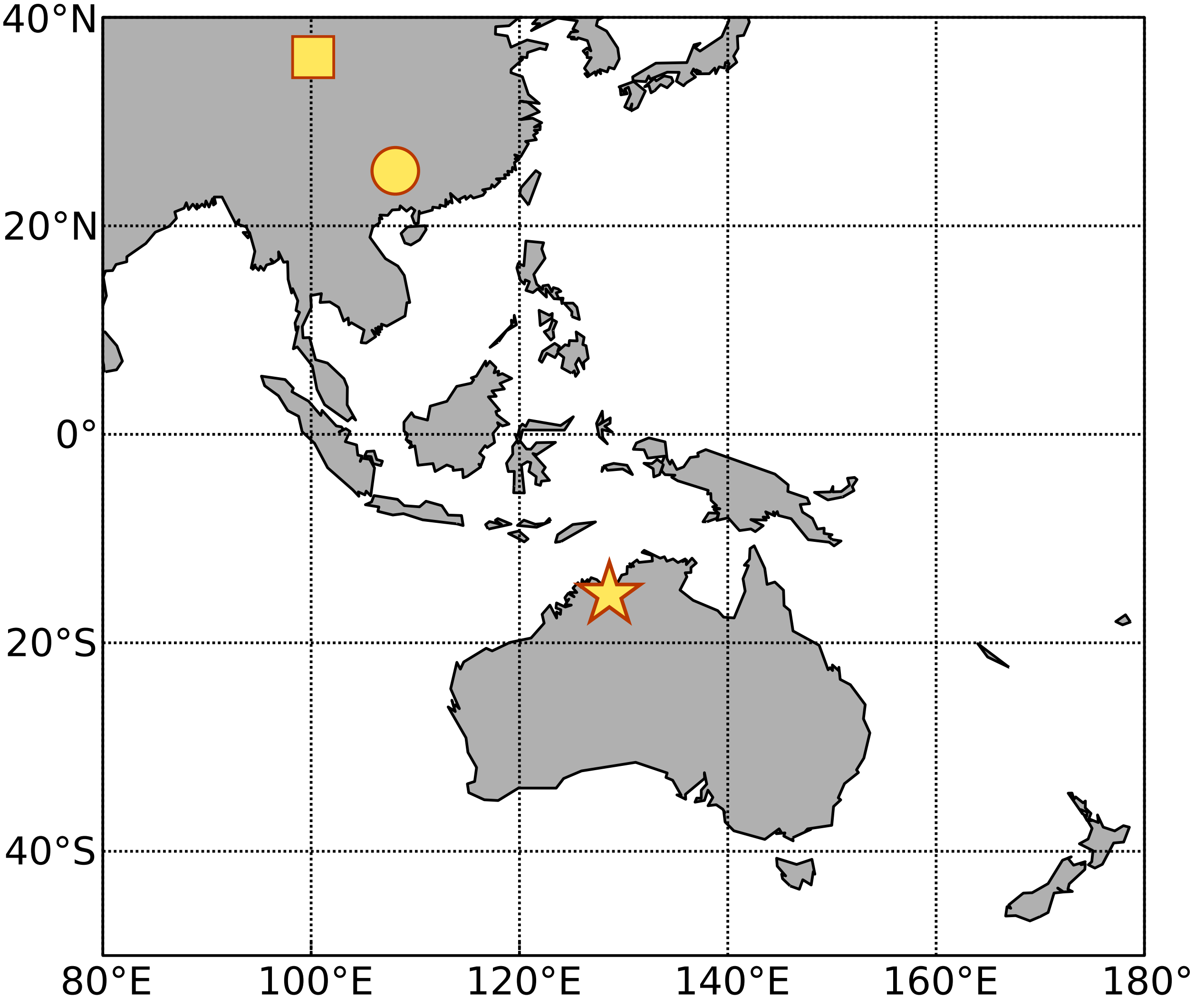

Supplementary Figure 2: **Location of the different proxies used in this study.** Dongge cave (circle), KNI-51 cave (star), and Qinghai Lake (square).

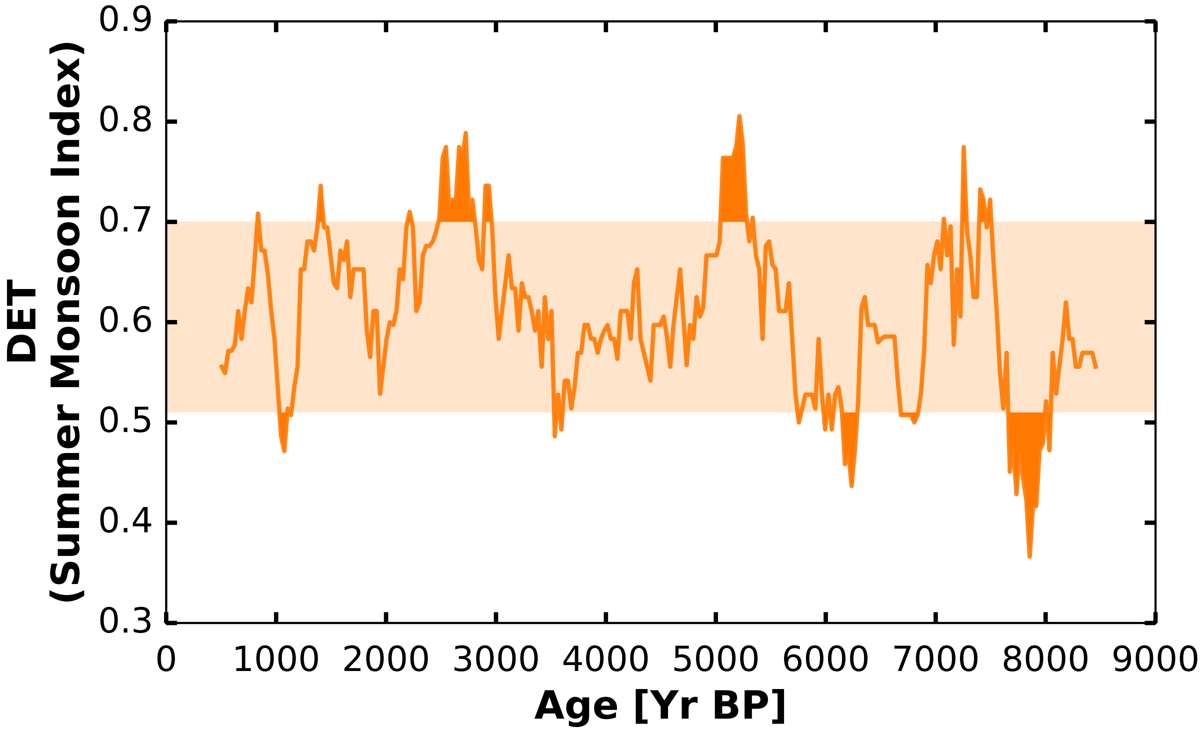

Supplementary Figure 3: **Determinism of the Summer Monsoon Index from Qinghai Lake.** Statistical significance is indicated by the horizontal band.

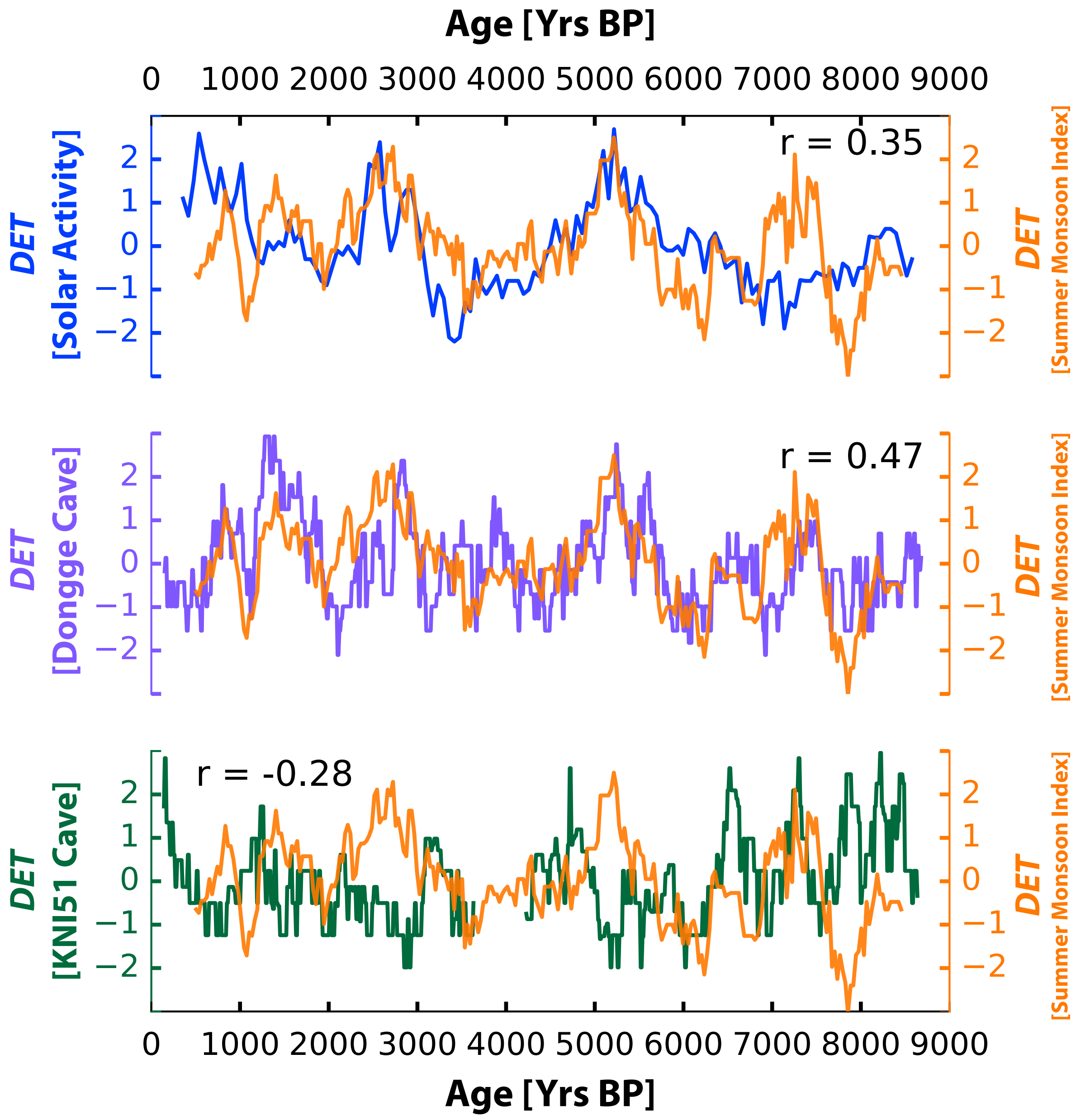

Supplementary Figure 4: **The analysis of SMI data from Qinghai Lake and its comparison with other proxies in the manuscript.** Solar activity, Dongge and KNI51 Caves.

South China			
**Regimes**	**Hu et al. (2008)**	**Our Findings**	
	**Heshang and Dongge Caves**	**Dongge Cave**	**Qinghai Lake**
**Strong Monsoon**	7.8-7.2 ka BP<br>6.2-6.1 ka BP<br>5.9-4.9 ka BP<br>4.0-3.9 ka BP<br>3.1-2.3 ka BP<br>2.1-1.3 ka BP<br>1.0-0.7 ka BP	5.6-5.2 ka BP<br>4.0-3.8 ka BP<br>3.0-2.6 ka BP<br>2.8-1.2 ka BP<br>1.0-0.7 ka BP	7.5-7.2 ka BP<br>5.35-5.0 ka BP<br>2.9-2.2 ka BP<br>1.4-1.3 ka BP<br>0.84-0.83 ka BP
**Weak Monsoon**	9.0-7.8 ka BP<br>7.2-6.2 ka BP<br>6.1-5.9 ka BP<br>4.9-4.0 ka BP<br>3.8-3.1 ka BP<br>2.3-2.1 ka BP<br>1.3-1.0 ka BP<br>0.7-0.0 ka BP	8.2-7.6 ka BP<br>7.2-6.8 ka BP<br>6.3-5.9 ka BP<br>4.6-4.1 ka BP<br>2.2-2.1 ka BP<br>1.2-1.0 ka BP<br>0.7-0.4 ka BP	8.1-7.6 ka BP<br>6.8-6.6 ka BP<br>6.2-5.9 ka BP<br>3.6-3.5 ka BP<br>1.15-1.0 ka BP

Supplementary Table 1: **Comparison of our and previous results.** Results for Southern China

North Australia			
**Regimes**	**Denniston et al. (2013)**	**McGowan et al. (2012)**	**Our Findings**
	**KNI-51**	**Pollen Data - Kimberley**	**KNI-51**
**Strong Monsoon**	9.0-7.0 ka BP<br>5.0-4.0 ka BP<br>1.2-0.9 ka BP	4.6-4.3 ka BP<br>1.3-1.0 ka BP	8.5-7.8 ka BP<br>7.4-7.1 ka BP<br>6.8-6.4 ka BP<br>4.9-4.6 ka BP<br>3.2-3.0 ka BP<br>1.3-1.1 ka BP
**Weak Monsoon**	6.3-4.5 ka BP<br>2.4-1.3 ka BP<br>1.0-0.7 ka BP	5.75-4.6 ka BP<br>3.20-2.8 ka BP<br>2.75-1.3 kaBP	7.6-7.5 ka BP<br>7.0-6.8 ka BP<br>6.2-5.0 ka BP<br>3.0-1.4 ka BP<br>0.8-0.6 ka BP

Supplementary Table 2: **Comparison of our and previous results.** Results for North Australia

Supplementary Discussion

Pre-processing paleoclimate proxy data

Paleoclimate proxy records commonly display long-term trends, such as in the Dongge Cave record [2]. Taking the difference between neighbouring data points in the sequence detrends the time series, and simultaneously transforms it such that it describes a different property of the underlying system. If we think of the original data set as corresponding to spatial 'positions' then this difference time series would give us the velocities. Such a method is common in time series analysis. In the paleoclimate context we are often faced with data sets with irregularly spaced time intervals between observations. We therefore employ the transformation cost function as our 'difference' metric. This not only detrends the data set, but produces a regularly spaced time series. We are now able to employ a number of well known techniques without having to account for unequal time steps.

Regional climate of Australian and Chinese proxy records

We use the high resolution speleothem paleoproxy records from cave KNI-51 (15.30°S, 128.61°E) in northwestern Australia and from Dongge Cave (DA) (25.28°N, 108.08°E) from southern China to outline the summer monsoon states of the last c. 9 ka. The details of the U/Th chronology and associated stable isotope records are provided by Denniston et al. [3] and Wang et al. [2] respectively. KNI-51 is located at the northern limits of Western Australia. Nearby Carlton Hill (15.49°S, 128.53°E) has a precipitation record extending back to 1897, with a mean annual rainfall over this period of 830mm (highest 1500mm/lowest 378mm) and an average 690 mm (83%) received during the monsoon season of December through to March [4]. Mean annual precipitation near Dongge Cave is 1753 mm with 80% of the rainfall falling during the monsoon season – May to October [5]. Both locations are well placed to capture the respective summer monsoon regimes located at the end points of the broader EAIASM system.

Summer Monsoon Index

In order to evaluate our findings we compare them with another proxy record from China, the summer monsoon index (SMI) (Supplementary Fig. 2). This index was derived from a ^{14}C-dated sediment record from Qinghai Lake (37°N, 100°E) [6]. The SMI time series is regularly sampled, therefore it is not necessary to apply TACTS, and the usual difference filter has been applied directly. As the two datasets in the manuscript, KNI-51 and DA, SMI is well placed to capture the respective summer monsoon regime. Quinghai lake is located at the northern end point of the broader EAIASM system and sensitive to EASM dynamics [6].

Our analysis of the lacustrine SMI strongly corroborates our results strongly and reveals alternating periods of statistically significant strong/weak monsoon activity states of centennial to millennial duration as in other proxies (see Supplementary Fig. 3 and Fig. 2 in main text). The shaded band in the figure depict the 90% confidence interval, with strong/weak monsoon states defined as exceeding these bands (see Recurrence plots, determinism and significance test section). Prolonged strong/weak states are clearly identified (Supplementary Tabs. 1 and 2). The direct comparison of SMI with DA, KNI-51, as well as with solar variation confirm our findings (Supplementary Fig.4).

Supplementary References

[1] Stuiver, M., Reimer, P.J. & Braziunas T.F. High-precision radiocarbon age calibration for terrestrial and marine samples. *Radiocarbon* **40**, 1127-1151 (1998).

[2] Wang, Y. *et al.* The Holocene Asian Monsoon: links to solar changes and North Atlantic climate. *Science* **308**, 854-857 (2005).

[3] Denniston, R. F. *et al.* A Stalagmite record of Holocene IndonesianAustralian summer monsoon variability from the Australian tropics. *Quaternary Science Reviews* **78**, 155-168 (2013).

[4] Bureau of Meteorology, Climate Data Online, http://www.bom.gov.au/climate/data/ (2013).

[5] Dykoski, C. A. *et al.* A high-resolution, absolute-dated Holocene and deglacial Asian monsoon record from Dongge Cave, China. *Earth and Planetary Science Letters* **233**, 71-86 (2005).

[6] An, Z. *et al.* Interplay between the Westerlies and Asian monsoon recorded in Lake Qinghai sediments since 32 ka. *Scientific Reports* **2**, 619 (2012).
